# Rare case of discrepant ABO typing between mother and newborn caused by para-Bombay AB phenotype

**DOI:** 10.1016/j.htct.2025.103742

**Published:** 2025-02-15

**Authors:** Tatiane Aparecida de Paula Vendrame, Giovana Faria Devides, Nayara Morais Silva, Marcela Custódio Mendes, Márcia Léia Morais Sampaio, Marcia Miyuki Nakamura, Greice Ap.Chetta de Klerk, Lilian Castilho, Afonso Cortez, Flavia Roche Moreira Latini, Carine Prisco Arnoni

**Affiliations:** a Associação Beneficente de Coleta de Sangue, Colsan, São Paulo, SP, Brazil; b Hospital Municipal e Maternidade Prof° Mario Degni, São Paulo, SP, Brazil; c Hemocentro, Unicamp, Campinas, SP, Brazil

## Introduction

The ABO system is the most important blood group in transfusion medicine. Humans have two types of alpha-(1,2)-fucosyltransferase encoded by *FUT1* and *FUT2* genes. *FUT1* encodes the H enzyme which determines the expression of the H type 2 antigen in the erythroid lineage, whereas *FUT2* encodes a Se enzyme which controls the expression of the H type 1 antigen in various secretory fluids. Therefore, the H type 2 antigen is the precursor structure for the formation of A and B antigens on red blood cells (RBCs), while in body fluids such as saliva, these antigens are produced from the H type 1 antigen.^1^

Deficient production of H antigens leads to rare phenotypes. The Bombay phenotype (H-) is caused by non-functional *FUT1* and *FUT2* genes, resulting in the complete absence of H, A, and B antigens on RBCs, and in saliva and body fluids, even though the individual inherits the normal ABO gene. The para-Bombay phenotype (*H* + ^w^) is characterized by low expression of the H antigen (and consequently, A and B antigens) on RBCs. It may arise from non-functional *FUT1* alleles but at least one functional *FUT2* allele which leads to H antigen production by the Se enzyme that is adsorbed from the serum into the RBCs. Additionally, the para-Bombay phenotype may result from a low H enzyme activity encoded by a weakly-functional *FUT1* allele.^2^

Phenotypes related to H-deficiency are extremely rare worldwide, but are more common in South Asian populations.^2^ The frequency of para-Bombay phenotype in Taiwanese populations is 1/8000 and Bombay phenotype is present in 1/10,000 in India, while it occurs in 1/1,000,000 in Europe.^2^^,^^3^ In Brazil, it is difficult to inform the frequency of these rare phenotypes since there are few reported cases. According to the Genome Aggregation Database (gnomAD), the frequency of the FUT1*02W.01 and FUT1*01N.09 alleles in Americans is 1/14,000 and 1/33,000, respectively.^4^

Here, we report a rare case of discrepant ABO typing between mother (typed as O) and her newborn (typed as AB) caused by an undetected para-Bombay AB phenotype. Additionally, we describe the complementary methodologies used to solve the case.

## Case report

The proband is a 36-year-old parturient typed as O in tube test (anti-A BIRMA-1, anti-B LB-2; Fresenius, São Paulo, Brazil) with no transfusion history or previous pregnancies. ABO typing was requested for the newborn and showed discordant results, as it was typed as AB. To exclude sampling, technical and transcription errors, second samples were requested but both results were confirmed. The father's sample was typed as A. Samples were sent to our reference laboratory for further investigation. The proband agreed to participate of this study and signed an informed consent form. The study was conducted in accordance with our institutional ethics review.

In the reference laboratory, ABO typing was performed using the DG Gel ABO/Rh(2D) gel test (Grifols, Barcelona, Spain) and the same results were found, with concordant reverse typing results and no weak reactions detected. The newborn's samples showed a negative direct antiglobulin test. The proband's sample was subjected to complementary serological and molecular tests, and an antibody against a high frequency antigen with a 3+ reaction was observed at room temperature, but it did not react at 37 °C. The RBCs were tested with anti-A1 lectin and anti-H lectin BSA® (both from Fresenius) and reactions were negative. Adsorption and elution tests with the anti-A1 (BIRMA-1) and anti-B (LB-2) commercial reagents revealed weak expression of both antigens. Lewis phenotyping showed Le(a-b-), and the ABH antigens inhibition test of saliva showed the presence of A, B and H antigens.

A molecular investigation was requested to conclude the case and confirm the serological findings. DNA sequencing was performed as previously reported.^5^ Sequencing of exons 6 and 7 of *ABO* revealed the presence of both ABO*A and ABO*B alleles. *FUT1* sequencing detected single nucleotide variants c.35C>T and c.269G>*T* in homozygosity, which characterize the FUT1*02W.01/FUT1*02W.01 genotype leading to weak H expression. Serological and molecular investigations confirmed the presence of the para-Bombay AB phenotype.

## Discussion

**Figure 1:**
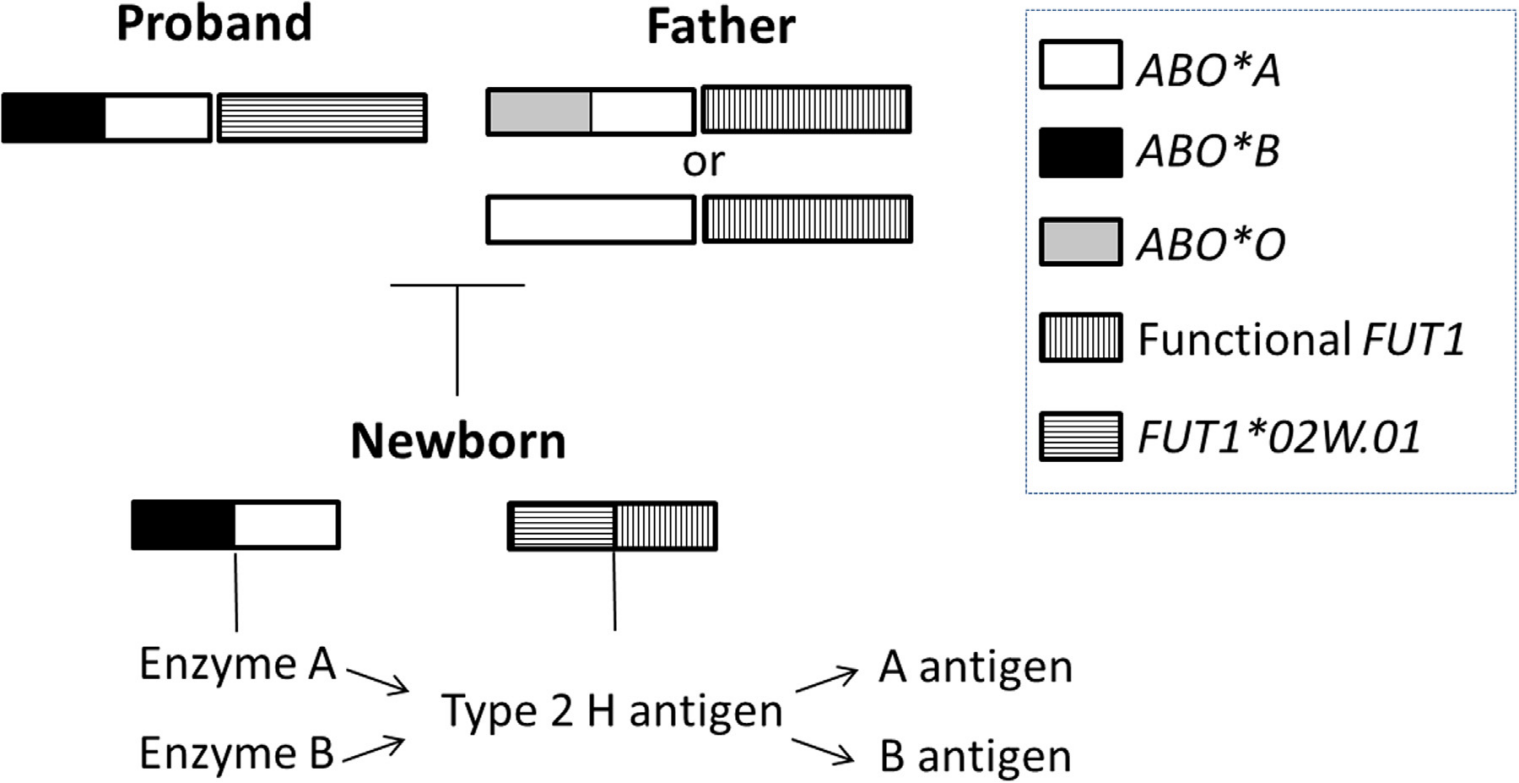
Schematic figure of genetic background involved in discrepant ABO typing between a mother and her newborn. The newborn inherited an ABO*B allele from the mother and an ABO*A from the father. Although the newborn inherited a *FUT*1 variant allele from his mother, he received a functional *FUT1* from his father that produces normal amounts of the H antigen, which is altered by both N-acetylgalactosaminyltransferase (enzyme A) and galactosyltransferase (enzyme B), producing A and B antigens. The genotypes of the father and newborn are deduced from the phenotype, since their samples have not been genotyped.

The present case reported a family study in which we observed discordant ABO typing between a mother and her newborn. Complementary serological studies and molecular investigation were used to accurately characterize the phenotypes and resolve the complex case of discrepancy between the mother and the newborn. According to the findings, the newborn inherited functional *FUT1* and ABO*A alleles from the father, and a variant of *FUT1* (FUT1*02W.01) and ABO*B alleles from the mother. The H antigen produced by the functional FUT1 was catalyzed by both N-acetylgalactosaminyltransferase and galactosyltransferase produced by the ABO alleles. Therefore, the newborn's expression of AB antigens was normal. A schematic figure of genetic background is shown in Figure 1.

The present study demonstrates that, as shown by other authors,^6^^,^^7^ the para-Bombay phenotype may remain undetected in standard routine blood group typing and may be mislabelled as blood group O if more detailed investigations are not performed. In contrast to the Bombay phenotype, para-Bombay individuals usually develop clinically insignificant anti-H and anti-HI antibodies^7^^,^^8^. Similarly, the proband had an antibody reacting only at room temperature, without thermal amplitude, which did not represent a risk to the newborn.

## Conflicts of interest

The authors declare that they have no conflicts of interest relevant to the manuscript.
